# Ensemble of decision tree reveals potential miRNA-disease associations

**DOI:** 10.1371/journal.pcbi.1007209

**Published:** 2019-07-22

**Authors:** Xing Chen, Chi-Chi Zhu, Jun Yin

**Affiliations:** School of Information and Control Engineering, China University of Mining and Technology, Xuzhou, China; University of Calgary, CANADA

## Abstract

In recent years, increasing associations between microRNAs (miRNAs) and human diseases have been identified. Based on accumulating biological data, many computational models for potential miRNA-disease associations inference have been developed, which saves time and expenditure on experimental studies, making great contributions to researching molecular mechanism of human diseases and developing new drugs for disease treatment. In this paper, we proposed a novel computational method named Ensemble of Decision Tree based MiRNA-Disease Association prediction (EDTMDA), which innovatively built a computational framework integrating ensemble learning and dimensionality reduction. For each miRNA-disease pair, the feature vector was extracted by calculating the statistical measures, graph theoretical measures, and matrix factorization results for the miRNA and disease, respectively. Then multiple base learnings were built to yield many decision trees (DTs) based on random selection of negative samples and miRNA/disease features. Particularly, Principal Components Analysis was applied to each base learning to reduce feature dimensionality and hence remove the noise or redundancy. Average strategy was adopted for these DTs to get final association scores between miRNAs and diseases. In model performance evaluation, EDTMDA showed AUC of 0.9309 in global leave-one-out cross validation (LOOCV) and AUC of 0.8524 in local LOOCV. Additionally, AUC of 0.9192+/-0.0009 in 5-fold cross validation proved the model’s reliability and stability. Furthermore, three types of case studies for four human diseases were implemented. As a result, 94% (Esophageal Neoplasms), 86% (Kidney Neoplasms), 96% (Breast Neoplasms) and 88% (Carcinoma Hepatocellular) of top 50 predicted miRNAs were confirmed by experimental evidences in literature.

## Author summary

MiRNAs are known as gene regulators and play critical roles in various biological processes. Many associations between miRNAs and human diseases have been identified, which promotes the understanding towards the molecular mechanisms of diseases and contributes to prevention and treatment of diseases. Computational methods of predicting potential miRNA-disease associations make the discovery more efficient and experiments more productive. We developed EDTMDA by constructing a computational framework integrating ensemble learning and dimensionality reduction. We performed global LOOCV, local LOOCV and 5-fold cross validation to evaluate performance of EDTMDA, which outperformed many classic methods. In addition, we carried out three types of case studies on important diseases, which were used to evaluate performance of model based on known associations in HMDD V2.0, for new diseases without known associations and based on known associations in HMDD V1.0. As a result, most predicted miRNAs in top 50 predictions were confirmed by experimental evidences in literature. So, we believe that EDTMDA can make reliable predictions and guide experiments to uncover more miRNA-disease associations.

## Introduction

MicroRNAs (miRNAs) are a kind of endogenous non-coding RNA with the length of about 22 nucleotides, regulating the expression of genes by base paring with target messenger RNA (mRNA) [1]. Since the first two miRNAs, lin-14 and let-7, both showing positive regulation for gene expression, were found [1], increasing new miRNAs have entered into researchers’ horizons. According to latest miRbase (Release 22), a miRNA database [2], 38589 entries representing hairpin precursor miRNAs and 48885 mature miRNA products in 271 species are collected. Accumulative evidences have revealed that miRNAs usually negatively regulate gene expression and they play critical roles in various biological processes such as cell proliferation, differentiation, aging and death [3–7]. In addition, mounting close relations between miRNAs and human diseases were confirmed by abundant experimental reports. For example, the existing study has validated that the expression of mir-140 was reduced in osteoarthritic cartilage [8]. Another example is that down-regulation of mir-145 was related to the increased expression of ERG, over-expression of which was the distinct characteristic of prostate cancer [9]. Besides, deregulation of a set of miRNAs including mir-150, mir-550, mir-124a, mir-518b and mir-539 was shown to be associated with transformation of gastritis into extranodal marginal zone lymphoma [10]. It is believed that uncovering more miRNA-disease associations gives an insight into molecular mechanisms of diseases and is favorable to diagnosis, prognosis and treatment of human complex diseases [11,12]. However, the existing knowledge of miRNA-disease associations is not enough and known associations were mostly obtained from previous biological experiments that were time-consuming and costly. Therefore, increasing studies were devoted to developing computational models to predict potential miRNA-disease associations [13]. These computational models could infer miRNAs that were more likely to be related to the given disease. Based on the prediction results, biological experiments were preferentially conducted for those miRNAs to improve experimental efficiency and save time as well as expenditure.

Base on the known miRNA-disease associations in some well-known biological databases such as HMDD V2.0 [14], dbDEMC [15] and miR2Disease [16], many computational models were proposed to predict associations between miRNAs and diseases, most of which were under the assumption that functionally similar miRNAs are likely to be associated with phenotypically similar diseases [17–19]. These methods cover two main categories, network algorithm and machine learning. For example, by integrating miRNA functional similarity network, the disease phenotype similarity network and the known disease-miRNA associations network, Jiang *et al*. [20] proposed initial computational model to uncover potential miRNA-disease associations. For an investigated disease *d*, each miRNA in the miRNA network was scored by the scoring function based on cumulative hypergeometric distribution. However, the model only considered local neighbor similarity information of each miRNA so that it did not show excellent prediction results. Xuan *et al*. [21] developed a model of Human Disease-MiRNA association Prediction (HDMP) to predict disease-related miRNAs. In this model, miRNA functional similarity was calculated and for miRNAs in the same family or cluster, their similarity scores were given higher weight because they tend to be associated with the same disease. For investigated disease *d*, relevance score of each miRNA candidate was calculated based on its most weighted *k* similar neighbors and then ranked to attained potential *d*-related miRNAs. Nevertheless, HDMP were unable to work for new disease without any known associated miRNAs. In addition, HDMP was also a local network similarity-based model that only considered miRNAs’ partial similarity information, such as neighbor information. In order to make full use of global network similarity information, Chen *et al*. [22] first adopted global network similarity measures and proposed a method of Random Walk with Restart for MiRNA-Disease Association prediction (RWRMDA) in which random walk was implemented on miRNA functional similarity network. Although the model achieved satisfactory prediction performance, it could not deal with new disease without any known associated miRNAs. Another model named MIDP was proposed by Xuan *et al*. [23] based on random walk on miRNA functional similarity network. Furthermore, MIDPE that was extended from MIDP could predict potential related miRNAs for new disease without any known related miRNAs. Chen *et al*. [24] proposed the model of Within and Between Score for MiRNA-Disease Association prediction (WBSMDA) to predict potential miRNA-disease associations, which specially calculated Gaussian interaction profile kernel similarity for diseases and miRNAs in addition to using the miRNA functional similarity and the disease semantic similarity. In this model, both of the Within-Score and Between-Score were defined from the view of miRNAs and diseases and the final association score for miRNA-disease pair was calculated by combining Within-Score and Between-Score. WBSMDA could also be effectively applied for new diseases and new miRNAs without any known associations. Chen *et al*. [25] further developed the model of Heterogeneous Graph Inference for MiRNA-Disease Association prediction (HGIMDA) in which the heterogeneous graph was constructed with the same inputs as WBSMDA. An iteration process was adopted based on the graph to infer potential miRNA-disease associations. For a further improvement of prediction accuracy, Chen *et al*. [26] proposed another method named Matrix Decomposition and Heterogeneous Graph Inference for miRNA-disease association prediction (MDHGI), which fully utilized matrix decomposition technique for known miRNA-disease associations before constructing the heterogeneous graph that was same as HGIMDA. In addition, method of Super-Disease and MiRNA for potential MiRNA–Disease Association prediction (SDMMDA) was proposed by Chen *et al* [27]. In order to improve the similarity measures of diseases and miRNAs, the model introduced ‘super-miRNA’ and ‘super-disease’ that were obtained by clustering as many as possible similar miRNAs or diseases. In addition, You *et al*. [18] proposed the prediction model of Path-Based MiRNA-Disease Association prediction (PBMDA) that integrated various biological datasets that was same as MDHGI into the heterogeneous graph. In the graph, the association possibility was calculated by summing all path scores between a miRNA and a disease. Specially, the model penalized long paths by a decay function because these paths were considered to make less contribution to the association score for the miRNA-disease pair. However, the distance-decay function in this model was relatively simple and could be further optimized. Yu *et al*. [28] proposed the prediction method, MaxFlow, which constructed a miRNAome-phenome network graph where a source node and a sink node were introduced. For the given disease, the maximum information flow from the source over all links to the sink were calculated and flow quantity leaving a miRNA node was used as the association score between the miRNA and the given disease. Furthermore, Chen *et al*. [29] developed another prediction model of Bipartite Network Projection for MiRNA–Disease Association prediction (BNPMDA). This model first constructed the bias ratings for miRNAs and diseases based on three networks, including the known miRNA–disease association network, the disease similarity network and the miRNA similarity network. Then bipartite network recommendation algorithm was implemented to reveal potential miRNA-disease associations.

In fact, many previous computational models were established based on other types of interaction networks, such as protein-protein interaction (PPI) network, miRNA-target interaction network and so on. For example, Shi *et al*. [30] developed prediction model by mapping disease genes and miRNA targets on PPI networks. For a given miRNA and disease, random walk was performed on the network using the disease genes and the miRNA targets as seeds simultaneously to obtain enrichment scores as association scores of the miRNA-disease pairs. Additionally, Mork *et al*. [31] proposed a model of miRNA-Protein-Disease (miRPD) association prediction with proteins as the mediators, which integrated miRNA–protein associations and protein–disease associations to predict novel associations between miRNAs and diseases. However, performance of miRPD was strongly limited by miRNA-target interactions with the high false positive rate. In addition, Pasquier *et al*. [32] established MiRAI model that represented distributional information of miRNAs and diseases in a high-dimensional vector space and predicted novel miRNA-disease associations in terms of vector similarities.

Nowadays, machine learning has been widely applied in biomedical research [33,34], such as drug target prediction [35], transcription factor binding prediction [36], functional variant annotation [37], synergistic drug combination prediction [38], small molecule-miRNA interaction prediction [39], association prediction between long non-coding RNAs and diseases [40], and disease related RNA methylation prediction [41]. Many machine learning-based methods have been proposed to infer potential miRNA-disease associations [13]. Unlike many previous models, the model of Matrix Completion for MiRNA-Disease Association prediction (MCMDA) developed by Li *et al*. [17] was only depended on known miRNA-disease associations where singular value thresholding (SVT) algorithm was used to conduct matrix completion procedure and predict new miRNA-disease association. The drawback of MCMDA was that it could not predict miRNAs for new diseases without any associations. Chen *et al*. [42] proposed a model named Restricted Boltzmann Machine for Multiple types of MiRNA-Disease Association prediction (RBMMMDA) to predicted not only novel miRNA-disease associations but also types of association. In RBMMMDA, a two-layer undirected graphical model of Restricted Boltzmann Machine (RBM) was constructed and trained to implement prediction. RBMMMDA also could not predict miRNAs for new diseases without any known association information. Xu *et al*. [43] proposed a method based on a heterogeneous MiRNA-Target Dysregulated Network (MTDN). A classifier named Support Vector Machine (SVM) was built to separate positive miRNA-disease associations from negative ones based on features extracted from MTDN. Nevertheless, it was difficult to select accurate negative samples because of unavailable validation for the negative ones. Another model named Regularized Least Squares for MiRNA-Disease Association prediction (RLSMDA) that did not need negative samples was developed by Chen *et al*. [44]. Under the framework of Regularized Least Squares (RLS), cost functions were defined and minimized to yield optimal classifiers from miRNA and disease sides, respectively. Then the weighted average strategy was adopted to combine two optimal classifiers to obtain final prediction results. Furthermore, Chen *et al*. [27] introduced the model of Ranking-based K-Nearest-Neighbors for MiRNA-Disease Association prediction (RKNNMDA) to infer potential associations between miRNAs and diseases. Based on *k*-nearest-neighbors for miRNAs and diseases, the model calculated Hamming loss to rank these neighbors with SVM and utilized weighted voting to each predicted miRNA-disease association. In addition, Chen *et al*. [45] proposed another model called Laplacian Regularized Sparse Subspace Learning for MiRNA-Disease Association prediction (LRSSLMDA) which achieved prediction scores from miRNA and disease side, respectively. The model’s inputs were miRNA/disease statistical features and graph theoretic features that were extracted from the miRNA/disease similarity. Then objective functions were built in miRNA/disease side with *L*_*1*_-norm constraint and Laplacian regularization terms. Final predictive results were attained by combining optimization results for objective functions. Furthermore, Chen *et al*. [46] developed the model of Predicting MiRNA–Disease Association based on Inductive Matrix Completion (IMCMDA), which was a matrix completion-based model. MiRNA-disease association matrix was a sparse matrix and missing association values of miRNA-disease pairs could be completed by means of miRNA similarity and disease similarity feature vectors.

Considering different limitations of previous models and improvement room for prediction accuracy, we developed the model of Ensemble of Decision Tree based MiRNA-Disease Association prediction (EDTMDA) to infer novel miRNA-disease associations. The inputs of the model were features which were extracted from integrated miRNA similarity, disease similarity and known miRNA-disease associations. The model adopted ensemble learning strategy that integrated multiple classifiers (base learners) to get final prediction results, which reflected association probability for candidate miRNA-disease pairs. Three cross validation methods, including global leave-one-out cross validation (LOOCV), local LOOCV and 5-fold cross validation (5-fold CV) were implemented to evaluate performance of EDTMDA. As a result, AUC of 0.9309 for global LOOCV, 0.8524 for local LOOCV and 0.9192+/-0.0009 for 5-fold CV were obtained. To our knowledge, the AUCs of EDTMDA are higher than almost all previous models. In addition, three types of case studies for important human diseases were further carried out to evaluate the ability to predict miRNAs related with the investigated disease. There were 47 (Esophageal Neoplasms), 43 (Kidney Neoplasms), 48 (Breast Neoplasms) and 44 (Carcinoma Hepatocellular) of top 50 predictions confirmed by previously published literature. These aforementioned validation experiments proved that EDTMDA is a reliable and excellent model to predict potential miRNA-disease associations.

## Materials and methods

### Human miRNA-disease associations

In our work, known human miRNA-disease associations verified by experimental evidences in literature were obtained from HMDD V2.0 which included 5430 associations between 495 miRNAs and 383 diseases [14]. Here, *Y* ∈ *R*^*nm*×*nd*^ was used to denote an adjacency matrix, where *nm* and *nd* represented the number of miRNAs and diseases, respectively. If miRNA *m*(*i*) and disease d(*j*) had association according to HMDD V2.0, the element *Y*(*m*(*i*), *d*(*j*)) equaled to 1, otherwise 0.

### MiRNA functional similarity

MiRNA functional similarity scores could be computed based on the MISIM method proposed by Wang *et al*. [47] and downloaded from the website: http://www.cuilab.cn/files/images/cuilab/misim.zip. We denoted *FS* as the score matrix of miRNA functional similarity and the element *FS*(*m*(*i*), *m*(*j*)) represented the functional similarity scores between miRNA *m*(*i*) and *m*(*j*).

### Disease semantic similarity model 1

Disease semantic similarity was computed according to the literature [47]. we download MeSH descriptors from the National Library of Medicine (http://www.nlm.nih.gov/), from which the relationship of various diseases could be obtained based on disease Directed Acyclic Graph (DAG). For example, a *DAG*(*D*) = (*D*,*T*(*D*), *E*(*D*)) was used to represent disease *D*, where T(*D*) was the node set including all parent nodes of disease *D* and disease *D* itself, and E(*D*) was defined as the set of edges pointing to child nodes from parent notes. In DAG(*D*), we defined the semantic value of disease *D* to *DV1*(*D*) as follows:

$$DV1\left(D\right)=\sum_{d\in T\left(D\right)}D1_{D}\left(d\right)$$ (1)

$$\left\{ \begin{array}{l} D1_{D}\left(d\right)=1\;\text{if}\;d=D\\ D1_{D}\left(d\right)=\text{max}\left\{\Delta *D1_{D}\left(d^{\prime }\right)|d^{\prime }\in \text{children}\;\text{of}\;d\right\}\;\text{if}\;d\neq D \end{array}\right.$$ (2)

where *D*1_*D*_(*d*) represented the contribution of disease *d* to the semantic value of disease *D* in DAG(*D*). As shown in Eq 2, disease *D* was the most specific disease in DAG(*D*) and its contribution to the semantic value of itself was set to 1. Those parent nodes locating farther from node *D* are more general denominations, having fewer contribution to the semantic value of disease *D*. To realize that, semantic contribution factor Δ was introduced (0< Δ <1) and we set Δ = 0.5 in this study, referring to the literature [47]. Based on the assumption that two diseases sharing larger parts in their DAGs tend to have higher semantic similarity, the semantic similarity between disease *d*(*i*) and *d*(*j*) could be defined as follows:

$$SS1\left(d\left(i\right),d\left(j\right)\right)=\frac{\sum_{t\in T\left(d\left(i\right)\right)\cap T\left(d\left(j\right)\right)}\left(D1_{d\left(i\right)}\left(t\right)+D1_{d\left(j\right)}\left(t\right)\right)}{DV1\left(d\left(i\right)\right)+DV1\left(d\left(j\right)\right)}$$ (3)

### Disease semantic similarity model 2

In order to obtain more comprehensive and accurate disease semantic similarity assessment, we needed to measure the similarity from different perspectives. Therefore, another model of measuring disease semantic similarity was adopted according to the literature [21]. We considered that the number of disease DAGs that a disease term may appear in are not always the same and for disease terms in the same layer of DAG(*D*), the disease term appearing in fewer DAGs should be more informative. i.e., the disease term should have larger semantic contribution to disease *D*. In this model, semantic contribution of disease *d* to disease *D* in DAG(*D*) was defined as follows:

$$D2_{D}(d)=-\text{log}\left[\frac{\text{the}\;\text{number}\;\text{of}\;\text{DAGs}\;\text{including}\;d}{\text{the}\;\text{number}\;\text{of}\;\text{diseases}}\right]$$ (4)

Similar to disease semantic similarity model 1, the semantic value of disease *D* and semantic similarity between disease *d*(*i*) and *d*(*j*) was respectively given as follows:

$$DV2\left(D\right)=\sum_{d\in T\left(D\right)}D2_{D}\left(d\right)$$ (5)

$$SS2\left(d\left(i\right),d\left(j\right)\right)=\frac{\sum_{t\in T\left(d\left(i\right)\right)\cap T\left(d\left(j\right)\right)}D2_{d\left(i\right)}\left(t\right)+D2_{d\left(j\right)}\left(t\right)}{DV2\left(d\left(i\right)\right)+DV2\left(d\left(j\right)\right)}$$ (6)

Two disease semantic similarity models defined semantic contributions of the disease *d* to disease *D* in DAG(D) in different ways. We defined it based on the theory that those parent nodes locating farther from node *D* are more general denominations, having fewer contribution to the semantic value of disease *D* in model 1, while in model 2, we defined it by considering that the disease appearing in fewer DAGs should be more special and have larger semantic contribution to disease *D*.

### Gaussian interaction profile kernel similarity

According to the literature [48], we could calculate Gaussian interaction profile kernel similarity for miRNAs (diseases), which constructed Gaussian kernel with the adjacency matrix *Y*. Taking miRNA as an example, the Gaussian interaction profile kernel similarity between miRNA *m*(*i*) and *m*(*j*) was calculated as follows:

$$GM\left(m\left(i\right),m(j)\right)=\text{exp}\left(-\gamma_{d}{\left\|Y\left(m\left(i\right),*\right)-Y\left(m\left(j\right),*\right)\right\|}^{2}\right)$$ (7)

Here, *Y*(*m*(*i*), *) and *Y*(*m*(*j*), *) are the *i*th and *j*th row of adjacency matrix *Y*, respectively, representing interaction information between corresponding miRNA and all diseases. Parameter *γ*_*d*_ controlled the bandwidth and was set as follows:

$$\gamma_{d}={\gamma^{\prime }}_{d}/\left(\frac{1}{nm}{\sum_{i=1}^{nm}\left\|Y\left(m_{i},*\right)\right\|}^{2}\right)$$ (8)

Analogically, according to the literature [48], Gaussian interaction profile kernel similarity for diseases could be calculated as follows:

$$GD\left(d\left(i\right),d(j)\right)=\text{exp}\left(-\gamma_{d}{\left\|Y\left(*,d\left(i\right)\right)-Y\left(*,d(j)\right)\right\|}^{2}\right)$$ (9)

$$\gamma_{d}={\gamma^{\prime }}_{d}/\left(\frac{1}{nd}{\sum_{i=1}^{nd}\left\|Y\left(*,d\left(i\right)\right)\right\|}^{2}\right)$$ (10)

where *Y*(*, *d*(*i*)) and *Y*(*, *d*(*j*)) are the *i*th and *j*th column of adjacency matrix *Y*, respectively, meaning interaction information between corresponding disease and all miRNAs.

### Integrated similarity for miRNAs and diseases

We computed disease semantic similarity based on DAGs of diseases, but we could not get DAGs for all diseases. That is, for the specific disease without DAG, the semantic similarity score between the disease and other diseases could not be computed in both disease semantic similarity models. In order to obtain all disease similarity information, we integrated disease semantic similarity with Gaussian interaction profile kernel similarity according to [24] as follows:

$$\begin{array}{l} SD\left(d\left(i\right),d\left(j\right)\right)\\ =\left\{ \begin{array}{l} \frac{SS1\left(d\left(i\right),d\left(j\right)\right)+SS2\left(d\left(i\right),d\left(j\right)\right)}{2}\;d\left(i\right)\;\text{and}\;d\left(j\right)\;\text{has}\;\text{semantic}\;\text{similarity}\\ GD\left(d\left(i\right),d\left(j\right)\right)\;\text{otherwise} \end{array}\right. \end{array}$$ (11)

where the average of two disease semantic similarity models was used as disease semantic similarity. Similarly, integrated miRNA similarity was given according to [24] as follows.

$$SM\left(m\left(i\right),m\left(j\right)\right)=\left\{ \begin{array}{l} FS\left(m(i),m(j)\right)\;m\left(i\right)\;\text{and}\;m\left(j\right)\;\text{has}\;\text{functional}\;\text{similarity}\\ GM\left(m\left(i\right),m\left(j\right)\right)\;\text{otherwise} \end{array}\right.$$ (12)

### EDTMDA

**Fig 1:**
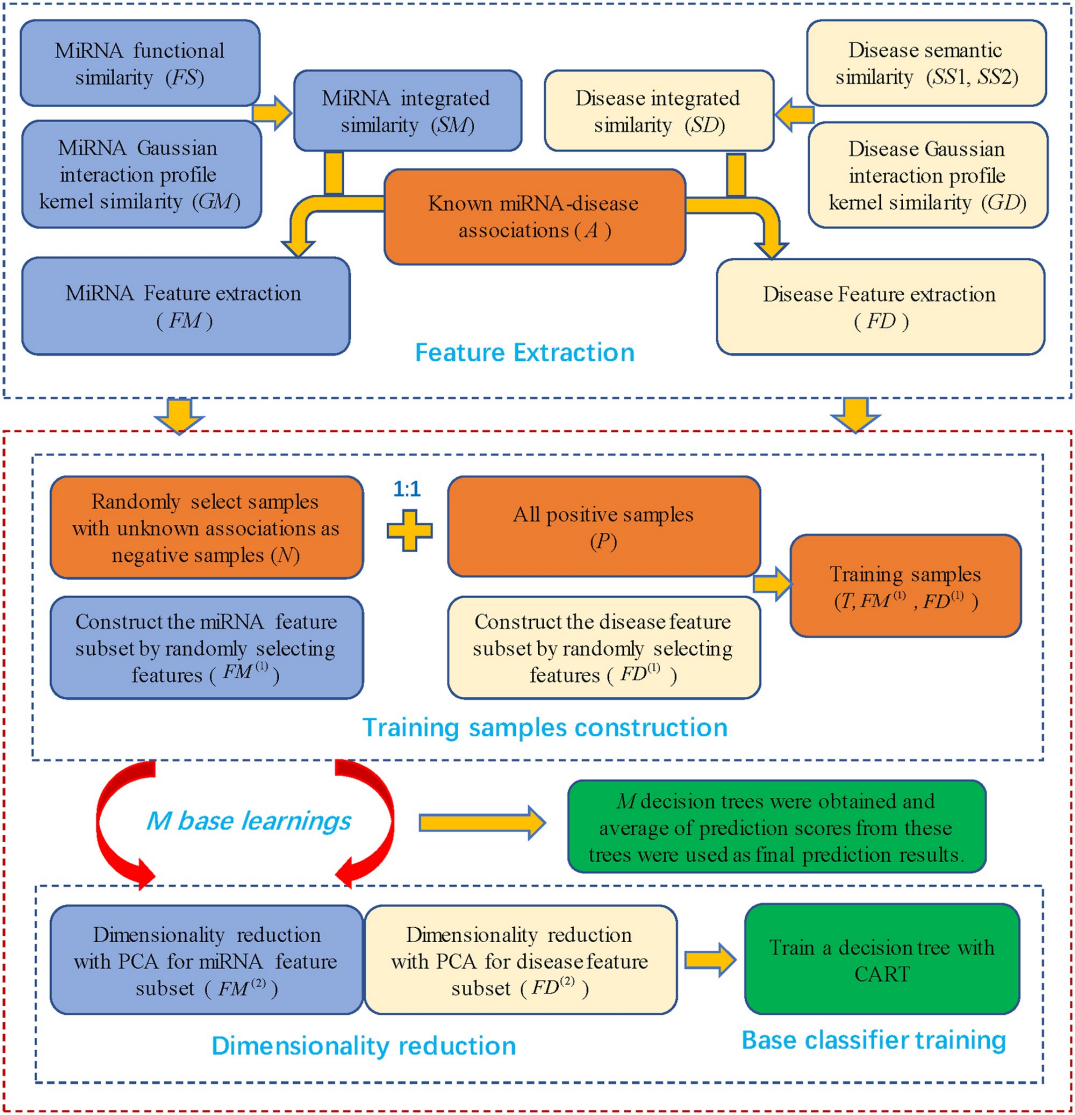
The flowchart of EDTMDA to predict miRNA-disease associations. MiRNA/disease features extracted from integrated miRNA/disease similarity and known miRNA-disease associations were inputs of our training model. *M* DTs were obtained from *M* base learnings and the average of prediction scores from all DTs were calculated as final prediction results.

EDTMDA was implemented based on integrated miRNA similarity matrix *SM*, integrated disease similarity matrix *SD* and known miRNA-disease associations matrix *Y*. At first, according to literature [49], three types of miRNA (disease) features were extracted based on the above matrixes *SM* (*SD*) and *Y* and used to form the feature vectors, represented by *FM (FD)*. Type 1 features covered the statistical measures summarized for each individual miRNA (disease) in *Y* and *SM* (*SD*) (including sum, mean, histogram distributions of miRNA/disease similarity scores); type 2 features included graph theoretical measures for network constructed by *SM* (*SD*) (including some neighbors’ attributes, betweenness, closeness, eigenvector centrality and Page-Rank scores of miRNA/disease similarity network); type 3 features focused on each miRNA-disease pair in *Y* based on matrix factorization of *Y* and graph theory-related statistics for network constructed by *Y*. Then, ensemble learning strategy was introduced based on random selection of negative samples and features, which included many base learnings and each base learning yield a base classifier, DT. Particularly, PCA was employed to reduce feature dimensionality during each base learning. The final association scores were obtained by computing the average of all prediction results from these DTs (motivated by the study of Ezzat *et al*. [50]). The base learning contained following steps (see Fig 1).

Firstly, construction of training sample set was operated. Because there were minority positive samples, accounting for about 2.9% of all possible samples in HMDD V2.0 used by our method, we chose all positive samples and some negative samples which were randomly singled out from the samples without known associations to construct the training set of our model. Particularly, negative samples were guaranteed to have the same number with positive samples. Here, *P* = {(*m*(*i*), *d*(*j*))|*Y*(*m*(*i*), *d*(*j*)) = 1} and *U* = {(*m*(*i*), *d*(*j*))|*Y*(*m*(*i*), *d*(*j*)) = 0} represented the set of positive samples and samples with unknown associations, respectively. The set *N* (*N*∈*U*) represented negative samples selected from *U* and |*N*| = |*P*| (|*N*| and |*P*| meant the number of elements in *N* and *P*, respectively). The set of *T* = *P* ⋃ *N* was training set in base learning. In addition, *FM*∈*R*^*nm*×*d*^ and *FD*∈*R*^*nd*×*d*^ (*d* represented the number of extracted miRNA/disease features) represented feature matrix of miRNAs and diseases in training set *T*, respectively. We constructed feature subsets of miRNAs and diseases by randomly selecting miRNA/disease features and used parameter *r* (0 < *r* ≤ 1) to control the size of feature subset. That is, ⌊*r*×*d*⌋ features were randomly sampled to construct feature subset. $FM^{\left(1\right)}\in R^{nm\times d_{1}}$ and $FD^{\left(1\right)}\in R^{nd\times d_{1}}$ represented feature subset of miRNAs and diseases, respectively (where *d*_1_ = ⌊*r*×*d*⌋).

Secondly, feature dimensionality reduction was applied to miRNA/disease feature subset. In our model, ensemble learning strategy was adopted to yield a large number of base learners, which brought much noise or redundant information to degrade prediction performance. To address this issue, PCA, an unsupervised dimensionality reduction algorithm [51], was employed to reduce miRNA/disease feature dimensionality of feature subset. Here, we saved top 10 miRNA (disease) features after dimensionality reduction, keeping almost all feature information. Here, *FM*^(2)^ and *FD*^(2)^ represented feature matrix of miRNAs and diseases after dimensionality reduction.

Thirdly, the DT, a base classifier, was trained with training set. For the sample in training set *T*, feature principle components of miRNA and disease, i.e., miRNA feature vector and disease feature vector in *FM*^(2)^ and *FD*^(2)^, were spliced as the feature vector of the sample, which was used as input vector of the DT. Our training set could also be denoted with *T*′ = {(*x*_1_, *y*_1_), (*x*_2_, *y*_2_), ⋯,(*x*_*n*_, *y*_*n*_)}, where $x_{i}=\left(x_{i}^{\left(1\right)},x_{i}^{\left(2\right)}\cdots ,x_{i}^{\left(d_{2}\right)}\right)$ was the *d*_*2*_-dimensional input vector (*d*_*2*_ = 20) and *y*_*i*_ represented the observed value of the *i*th sample in adjacency matrix *Y*, and *n* was the number of samples in training set. For the DT, we constructed the regression tree model with the arithmetic of CART, which was on the basis of squared error minimum criterion [52]. Yielding the regression tree could be described as a progress of building a binary decision tree recursively. If we selected the feature value $x_{i}^{\left(j\right)}$ to partition feature space *R*, *j* and *s* ($x_{i}^{\left(j\right)}=s$) were the splitting variable and splitting point, respectively, and two subspaces were defined as follows:

$$R_{1}\left(j,s\right)=\{x|x^{\left(j\right)}\leq s\}\;\text{and}\;R_{2}\left(j,s\right)=\{x|x^{\left(j\right)}>s\}$$ (13)

Then regression tree could be described as:

$$f\left(x\right)=c_{k}\;x\in R_{k},k=1,2$$ (14)

where *c*_*k*_ denoted output value of subspace *R*_*k*_ and its optimal value was calculated by minimizing squared error ${\sum_{x_{i}\in R_{k}}\left(y_{i}-f\left(x_{i}\right)\right)}^{2}$. The solution was given as follows:

$${\hat{c}}_{k}=\frac{1}{N_{k}}\sum_{x_{i}\in R_{k}\left(j,s\right)}y_{i}\;x\in R_{m},m=1,2$$ (15)

where *N*_*k*_ was the number of input vectors in subspace *R*_*k*_. In order to choose the optimal splitting variable and splitting point, variable *j* and *s* were traversed to solve the following equation:

$$\underset{j,s}{\text{min}}\left[{\sum_{x_{i}\in R_{1}\left(j,s\right)}\left(y_{i}-c_{1}\right)}^{2}+{\sum_{x_{i}\in R_{2}\left(j,s\right)}\left(y_{i}-c_{2}\right)}^{2}\right]$$ (16)

The optimal splitting variable *j*′ and splitting point *s*′ was obtained. The pair (*j*′, *s*′) was used to partition the feature space according to the formula (13) and the output was calculated based on the formula (14) and (15). Then new optimal splitting variable and splitting point were sought in subspace *R*_1_ and *R*_2_, respectively. Then new output ${\hat{c}}_{k}$ (*k* = 1,2,3,4) was calculated in 4 subspaces, respectively. This procedure was repeated until the subspace could not be partitioned. At last, the feature space was divided into *K* subspaces and the final regression tree was described as follows:

$$f\left(x\right)=c_{k}\;x\in R_{k},k=1,2,\cdots ,K$$ (17)

**Fig 2:**
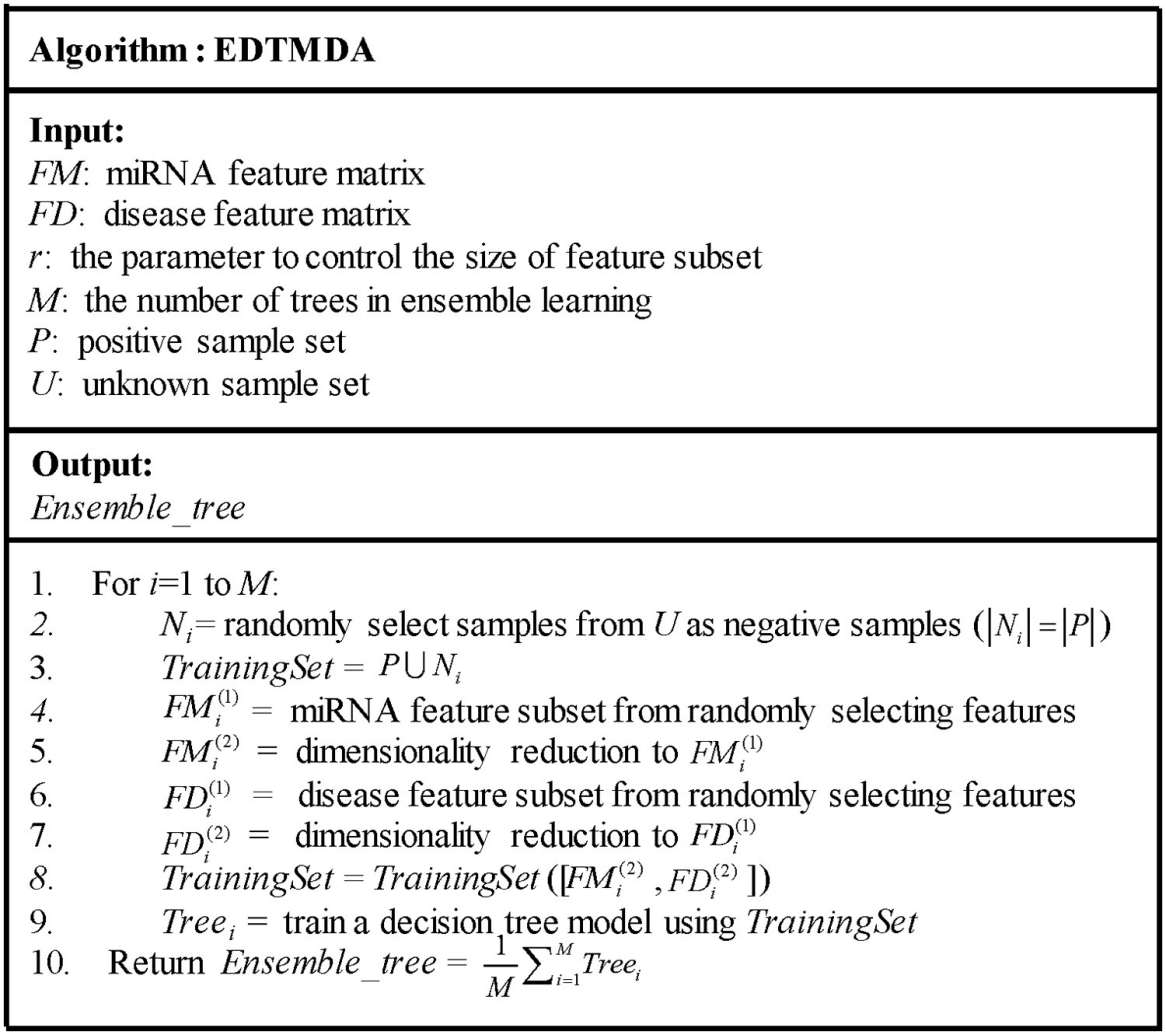
The pseudocode of EDTMDA to predict miRNA-disease associations.

Based on random selection of negative samples and miRNA/disease features, *M* base learnings including above three steps were implemented to yield *M* DTs. The simple average strategy was adopted for these DTs to obtain final prediction scores. Fig 2 shows the pseudocode of EDTMDA. The code and data of EDTMDA is freely available at https://github.com/chi-young1/EDTMDA.

## Results

### Performance evaluation

Based on known miRNA-disease associations in HMDD V2.0, we implemented LOOCV and 5-fold CV to evaluate the performance of EDTMDA. Receiver operating characteristic (ROC) curves are widely used to evaluate model performance in previous literature of predicting miRNA-disease associations and in order to more conveniently implement performance comparison, we also employed it in our study. Moreover, ROC curves are insensitive to class imbalance, which is suitable for assessing our model’s ability to recover hidden known associations from mass candidates (unknown associations).

LOOCV, including global LOOCV and local LOOCV, were implemented to evaluate the performance of EDTMDA. Global LOOCV was used to evaluate model’s global prediction ability for all disease simultaneously, which evaluated recover ability for a hidden miRNA-disease association from all unknown associations. Local LOOCV was used to evaluate model’s local prediction ability for a specific disease, which assessed the recover ability for a hidden miRNA-disease association from unknown associations of the investigated disease. Therefore, there is big difference for these two types of LOOCV. In global LOOCV, each known miRNA-disease association was singled out as test sample in turn and other known associations were treated as training samples for model training. Note that we recalculated Gaussian interaction profile kernel similarity of miRNAs and diseases when a known miRNA-disease association was removed, changing miRNA-disease adjacency matrix. Prediction scores of the test sample and all candidate samples (That is, those miRNA-disease pairs without association evidences) could be obtained after implementing EDTMDA. Then the test sample was ranked with all candidate samples based on their scores, and if the rank was higher than the specific threshold, the test sample was successfully predicted. Different from global LOOCV considering all diseases simultaneously, the test sample was only ranked with candidate samples containing the same disease as the test sample. In model performance evaluation, true positive rate (TPR, sensitivity) and false positive rate (FPR, 1-specificity) are usually calculated based on given threshold. Sensitivity indicates the percentage of the test samples ranked higher than the specific threshold; specificity means the percentage of negative samples ranked below the threshold. When different thresholds were given, we can obtain corresponding TPR and FPR to plot the ROC curve with the TPR as the vertical axis and FPR as the horizontal axis. ROC curve could be used to vividly show predictive performance of the model, and a ROC curve closer to the upper left corner of the figure represents more accurate performance. Furthermore, area under the ROC curve (AUC) was calculated to quantitatively evaluate model performance. AUC = 1 represents that the model has perfect prediction performance and AUC = 0.5 refers to random performance.

We compared the performance of EDTMDA with other classical models in terms of AUC under cross validation. The details of compared models were provided as follows: **HGIMDA** [25]: The model constructed a heterogeneous graph by integrating multiple biological data, where all paths with the length equal to three were summarized to infer potential miRNA-disease associations (The parameter used for comparison was *α* = 0.4). **MDHGI** [26]: The model employed matrix decomposition for miRNA-disease association matrix before implementing the heterogeneous graph inference that was same as HGIMDA (The parameters used for comparison were *α* = 0.1, *μ* = 10^−4^, max_*μ*_ = 10^10^, *ρ* = 1.1, *ε* = 10^−6^ and *α* = 0.4). **RLSMDA** [44]: The method combined two classifiers trained from the miRNA space and disease space respectively based on the framework of regularized least squares algorithm (The parameters used for comparison were *η*_*M*_ = 1, *η*_*D*_ = 1 and *ω* = 0.9). **HDMP** [21]: The relevance scores of unlabeled miRNAs were computed based on functional similarity of miRNAs’ *k* nearest neighbors. Besides, the members in the same miRNA family or cluster are assigned higher weight (The parameters used for comparison were *α* = 4, *β* = 4 and *k* = 20). **WBSMDA** [24]: The model defined the Within-Score and Between-Score from the miRNA side and disease side, then combined these score to infer potential miRNA-disease associations. **RWRMDA** [22]: Random walk was implemented on the miRNA-miRNA functional similarity network (The parameters used for comparison were *r* = 0.2 and threshold = 10^−6^). **MCMDA** [17]: The model utilized the matrix completion algorithm to update the adjacency matrix of known miRNA-disease associations (The parameters used for comparison were *ε* = 10^-4^ and max_iter = 500). **MiRAI** [32]: The model represented distributional information on miRNAs and diseases in a high-dimensional vector space and defined associations between miRNAs and diseases in terms of their vector similarity (The parameter used for comparison was *r* = 400). **MaxFlow** [28]: A combinatorial prioritization algorithm was designed for miRNA-disease association prediction by modifying the existing maximizing information flow method (The parameters used for comparison were *α* = 0.1, *β* = 0.6, *γ* = 100, *η* = 6 and *σ* = 10). **PBMDA** [18]: The model constructed a heterogeneous graph consisting of three interlinked sub-graphs and computed the accumulative contributions from all paths between a miRNA-disease pair as the association score, which specially set decay factor to cut down the contributions of longer paths to miRNA-disease association scores (The parameters used for comparison were *T* = 0.5, *L* = 3 and *α* = 2.26). **LRSSLMDA** [45]: A common subspace for the miRNA/disease profiles, a *L*_*1*_-norm constraint and Laplacian regularization terms were joint to construct the prediction model (The parameters used for comparison were *γ* = 2, *μ* = 1 and *λ* = 1). **MIDP** [23]: A novel random walk with different transition weight for labeled nodes and unlabeled nodes was implemented on miRNA functional similarity network to predict miRNAs related to the disease with some known related miRNAs and for the new disease without any known related miRNAs, the model extend the walking on a miRNA-disease bilayer network (The parameters used for comparison were *r*_*Q*_ = 0.4 and *r*_*U*_ = 0.1.).

**Fig 3:**
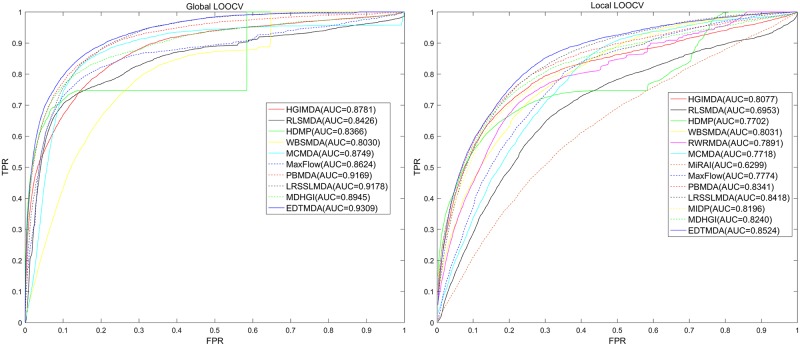
Performance comparisons between EDTMDA and other 12 prediction models (HGIMDA, RLSMDA, HDMP, WBSMDA, RWRMDA, MCMDA, MIDP, PBMDA, MaxFlow, LRSSLMDA, MiRAI and MDHGI) in terms of ROC curve and AUC based on local and global LOOCV, respectively. As a result, EDTMDA obtained AUCs of 0.9309 and 0.8524 in the global and local LOOCV, which exceed all of the above previous classical models.

Fig 3 showed the performance comparisons between EDTMDA and other several models in the framework of global and local LOOCV. EDTMDA, LRSSLMDA, PBMDA, MDHGI, HGIMDA, MCMDA, MaxFlow, RLSMDA, HDMP and WBSMDA obtained AUC of 0.9309, 0.9178, 0.9169, 0.8945, 0.8781, 0.8749, 0.8624, 0.8426, 0.8366 and 0.8030 in global LOOCV, respectively; they obtained 0.8524, 0.8418, 0.8341, 0.8240, 0.8077, 0.7718, 0,7774 0.6953, 0.7702 and 0.8031 in global LOOCV, respectively. RWRMDA and MIDP did not have an AUC value in global LOOCV because they could not simultaneously make predictions for all diseases. Additionally, global LOOCV also could not be implemented for MiRAI because the association scores yielded from the model were highly related with the number of known associated miRNAs of a disease. For a disease with more related miRNAs, the association scores for its candidate miRNAs were more likely to be higher. Therefore, it was not objective to simultaneously consider association scores of all diseases in global LOOCV. AUCs of 0.7891 for RWRMDA, 0.8196 for MIDP and 0.6299 for MiRAI were obtained in local LOOCV. Higher AUC values of EDTMDA in LOOCV indicated that our model had more accurate prediction than most previous models.

We implemented 5-fold CV to further evaluate the prediction performance of EDTMDA. In 5-fold CV, all positive samples (That is, those miRNA-disease pairs with known associations) were randomly divided into five equal-sized groups, four of which, along with same size of selected randomly negative samples, used to training the classifier. The omitted group (hidden positive samples) was added to all unknown associations to construct all candidates. Specially, we recalculated the Gaussian interaction profile kernel similarity of miRNAs and diseases when each group of miRNA-disease associations were removed. Then similar to global LOOCV, the association scores of candidates were calculated and then ranked by their scores. The higher the hidden positive samples were ranked, the better the performance was. That is, we removed some known associations and assessed ability to recover these hide associations to evaluate performance of model. This procedure was repeated 100 times because sample division was random in 5-fold CV. As a result, EDTMDA obtained average AUC with standard deviation of 0.9192+/-0.0009, surpassing all other methods compared (See Table 1), which further shows the superior performance of EDTMDA.

**Table 1:** AUC results between EDTMDA and other methods under 5-fold CV.

Methods	AUC
EDTMDA	0.9192+/-0.0009
LRSSLMDA	0.9181+/-0.0004
PBMDA	0.9172+/-0.0007
MDHGI	0.8794+/-0.0021
MCMDA	0.8767+/-0.0011
MaxFlow	0.8579+/-0.001
RLSMDA	0.8569+/-0.0020
HDMP	0.8342+/-0.0010
WBSMDA	0.8185+/-0.0009

### Dimensionality reduction effect analysis

In our method, multiple base learnings were constructed to generate many base classifiers (DTs) base on random selection of negative samples and miRNA/disease features, which also brought some noise or redundancy to influence final prediction results. To address this issue, we used PCA to implement dimensionality reduction for miRNA/disease feature subset. To evaluate the effect of dimensionality reduction to our model, we assessed performance of the method after removing dimensionality reduction step in each base learning. That is, we spliced miRNA and disease features of feature subset as the input of base classifiers. The AUC comparison results between EDTMDA with dimensionality reduction and EDTMDA without dimensionality reduction were shown in Table 2, which indicated that dimensionality reduction in base learning contributed to improve prediction performance of the model.

**Table 2:** AUC results of EDTMDA between with dimensionality reduction and without dimensionality reduction under three cross validations.

Methods	Global LOOCV	Local LOOCV	5-fold CV
EDTMDA with PCA	0.9309	0.8524	0.9192+/-0.0009
EDTMDA without PCA	0.9216	0.8423	0.9076+/-0.0012

### Comparison between EDTMDA and Random Forest (RF)

We conducted comparison of prediction performance between EDTMDA and RF which is also an ensemble learning method with DT as base classifier. Extracted miRNA features and disease features were spliced as the input vector of RF and RF was implemented using *RandomForestRegressor* that is an algorithm package of RF in Python, where default parameter values were used other than *n_estimators* (It was set as 50, meaning that the number of trees in RF is same as in EDTMDA). As shown in Table 3, EDTMDA is notably outperformed RF under three cross validations. There are two main differences between EDTMDA and RF. First, EDTMDA randomly selected a different negative sample set for each base classifier while RF performed bagging on the same negative set. That is, EDTMDA used more negative samples for model training than RF. Second, EDTMDA included all positive samples in training set for each base classifier, but RF performed bagging on the positive samples so that each DT in RF used only a subset of all positive samples. We concluded that prediction performance of the model was sensitive to positive samples and the best strategy was to include all positive samples for each base classifier in ensemble learning. Moreover, EDTMDA incorporated more data for model training, obtaining better prediction performance than RF.

**Table 3:** AUC results between EDTMDA and RF under three cross validations.

Methods	Global LOOCV	Local LOOCV	5-fold CV
EDTMDA	0.9309	0.8524	0.9192+/-0.0009
RF	0.8464	0.7745	0.8341+/-0.0035

### Case studies

To further access the prediction ability of EDTMDA, three types of case studies were carried out. For the sake of brevity, we selected several important human diseases to analyze in detail. The first type of case study was concerned with Esophageal Neoplasms and Kidney Neoplasms, and known miRNA-disease associations in HMDD V2.0 were used as training samples. All candidate miRNAs that were unassociated with the investigated disease in HMDD V2.0 were ranked according to their predicted association scores. Top 50 of candidate miRNAs were validated in two other miRNA-disease association databases, dbDEMC [15] and miR2Disease [16].

Esophageal Neoplasms is a serious malignancy with high mortality rate, ranking sixth among all cancer in mortality [53]. Squamous cell carcinoma (SCC) is the most common type of Esophageal Neoplasms and the black with SCC was three times higher than the white [54]. There will be 17190 new cases in Esophageal Neoplasms and 15850 people dying of the Esophageal Neoplasms in 2018 according to the study [55]. Many previous studies have confirmed the associations between the Esophageal Neoplasms and various miRNAs. For example, the higher expression of miRNA-506 was found in squamous cell carcinoma (ESCC) patients than in heathy people [56]. Moreover, according to the study [57], the expression of miRNA-382-5p notably increased and miRNA-133a-3p notably decreased in esophageal adenocarcinoma (EAC). In case study of Esophageal Neoplasms, 10 out of top 10 and 47 out of top 50 predicted miRNAs related to Esophageal Neoplasms were confirmed by dbDEMC or miR2Disease (See Table 4).

**Table 4:** EDTMDA was implemented to predict potential miRNAs related to Esophageal Neoplasms based on known associations in HMDD V2.0. The top 50 predicted miRNAs were verified in dbDEMC and miR2Disease. The first column records top 1–25 related miRNAs and the third column records the top 26–50 related miRNAs.

miRNA	evidence	miRNA	evidence
hsa-mir-106b	dbDEMC	hsa-mir-142	dbDEMC
hsa-mir-200b	dbDEMC	hsa-mir-195	dbDEMC
hsa-mir-16	dbDEMC	hsa-mir-218	unconfirmed
hsa-mir-18a	dbDEMC	hsa-mir-204	unconfirmed
hsa-mir-125b	dbDEMC	hsa-let-7d	dbDEMC
hsa-mir-221	dbDEMC	hsa-mir-29a	dbDEMC
hsa-mir-106a	dbDEMC	hsa-mir-146b	dbDEMC
hsa-mir-9	dbDEMC	hsa-mir-181b	dbDEMC
hsa-mir-222	dbDEMC	hsa-mir-199b	dbDEMC
hsa-mir-107	dbDEMC and miR2Disease	hsa-mir-138	unconfirmed
hsa-let-7e	dbDEMC	hsa-let-7i	dbDEMC
hsa-mir-125a	dbDEMC	hsa-mir-335	dbDEMC
hsa-mir-7	dbDEMC	hsa-mir-302c	dbDEMC
hsa-mir-182	dbDEMC	hsa-mir-181a	dbDEMC
hsa-mir-429	dbDEMC	hsa-mir-139	dbDEMC
hsa-mir-29b	dbDEMC	hsa-mir-20b	dbDEMC
hsa-mir-302b	dbDEMC	hsa-let-7g	dbDEMC
hsa-mir-30a	dbDEMC	hsa-mir-30c	dbDEMC
hsa-mir-1	dbDEMC	hsa-mir-17	dbDEMC
hsa-mir-127	dbDEMC	hsa-mir-135a	dbDEMC
hsa-mir-10b	dbDEMC	hsa-mir-19b	dbDEMC
hsa-mir-93	dbDEMC	hsa-mir-219	unconfirmed
hsa-mir-24	dbDEMC	hsa-mir-372	dbDEMC
hsa-mir-194	dbDEMC and miR2Disease	hsa-mir-224	dbDEMC
hsa-mir-32	dbDEMC	hsa-mir-30d	dbDEMC

Kidney Neoplasms, also known as Renal cell carcinoma (RCC), accounts for 2–3% of all the adult cancers [58]. It has been estimated that 65340 Americans will be diagnosed with Kidney Neoplasms and 14970 will die of the disease in 2018 [55]. Some studies have confirmed that dysregulation of miRNAs is closely related to Kidney Neoplasms. For example, Arai *et al*. [59] found that low expression of mir-10a-5p had association with overall survival in Kidney Neoplasms patients because downregulation of mir-10a-5p inhibited cancer cell migration and invasion. Another study showed that mir-21 played an important role in Kidney Neoplasms progression and could resist chemotherapeutic drugs used for treatment of Kidney Neoplasms [60]. As a result of case study for Kidney Neoplasms, 9 out of the top 10 and 43 out of the top 50 miRNAs were validated to have associations with Kidney Neoplasms by dbDEMC and miR2Disease (See Table 5).

**Table 5:** EDTMDA was implemented to predict potential miRNAs related to Kidney Neoplasms based on known associations in HMDD V2.0. The top 50 predicted miRNAs were verified in dbDEMC and miR2Disease. The first column records top 1–25 related miRNAs and the third column records the top 26–50 related miRNAs.

miRNA	evidence	miRNA	evidence
hsa-mir-16	dbDEMC	hsa-mir-1	dbDEMC
hsa-let-7a	dbDEMC	hsa-mir-92a	unconfirmed
hsa-mir-150	dbDEMC and miR2Disease	hsa-let-7i	dbDEMC
hsa-mir-200a	dbDEMC	hsa-mir-18a	dbDEMC
hsa-mir-155	dbDEMC	hsa-mir-210	dbDEMC and miR2Disease
hsa-mir-182	dbDEMC and miR2Disease	hsa-mir-296	unconfirmed
hsa-mir-125b	unconfirmed	hsa-mir-196a	dbDEMC
hsa-mir-34a	dbDEMC	hsa-let-7g	dbDEMC
hsa-mir-17	miR2Disease	hsa-mir-19a	dbDEMC
hsa-mir-146a	dbDEMC	hsa-mir-199a	dbDEMC and miR2Disease
hsa-mir-145	dbDEMC	hsa-mir-133a	unconfirmed
hsa-let-7c	dbDEMC	hsa-mir-29b	dbDEMC and miR2Disease
hsa-mir-9	dbDEMC	hsa-mir-19b	dbDEMC and miR2Disease
hsa-mir-367	unconfirmed	hsa-mir-25	dbDEMC
hsa-let-7b	unconfirmed	hsa-mir-223	dbDEMC
hsa-mir-29a	dbDEMC and miR2Disease	hsa-mir-106b	dbDEMC and miR2Disease
hsa-mir-181a	dbDEMC	hsa-mir-146b	dbDEMC
hsa-mir-222	dbDEMC	hsa-mir-193b	dbDEMC
hsa-mir-221	unconfirmed	hsa-mir-302c	unconfirmed
hsa-mir-203	dbDEMC	hsa-mir-99a	dbDEMC
hsa-mir-126	dbDEMC and miR2Disease	hsa-mir-195	dbDEMC
hsa-let-7d	dbDEMC	hsa-mir-205	unconfirmed
hsa-mir-199b	dbDEMC	hsa-mir-148a	dbDEMC
hsa-mir-200b	dbDEMC and miR2Disease	hsa-mir-130a	dbDEMC
hsa-let-7f	dbDEMC and miR2Disease	hsa-mir-181b	dbDEMC

We exhibited complete prediction results inferring potential disease-associated miRNAs that were ranked based on their predicted association scores, which we expect to be beneficial for experimental studies in the future (See S1 Table).

The second type of case study for Breast Neoplasms was implemented to prove the applicability of EDTMDA to new diseases without known related miRNAs. We removed all known Breast Neoplasms-miRNA associations in HMDD V2.0 so Breast Neoplasms could be regarded as new disease. After implementing EDTMDA to predict and rank potential Breast Neoplasms-related miRNAs based on other known disease-miRNA associations, we confirmed that 10 out of top 10 and 48 out of top 50 predicted Breast Neoplasms-related miRNAs were validated by HMDD V2.0, dbDEMC and miR2Disease (See Table 6). Hsa-mir-210, ranking first in our prediction result list, had the greatest possibility associating with Breast Neoplasms. The study of Zehentmayr *et al*. [61] has revealed the association that hsa-mir-210 was overexpressed in contralateral unaffected breasts (CUB) of patients with breast cancer. This case study showed that our model was also reliable when applied to predict miRNAs related with new diseases.

**Table 6:** EDTMDA was implemented to predict potential miRNAs associated with Breast Neoplasms as a new disease by removing all known associations containing Breast Neoplasms in HMDD V2.0 database. The top 50 predicted miRNAs were verified in dbDEMC, miR2Disease and HMDD V2.0. The first column records top 1–25 related miRNAs and the third column records the top 26–50 related miRNAs.

miRNA	evidence	miRNA	evidence
hsa-mir-210	dbDEMC;miR2Disease; HMDD V2.0	hsa-mir-155	dbDEMC;miR2Disease; HMDD V2.0
hsa-mir-31	dbDEMC;miR2Disease; HMDD V2.0	hsa-mir-15a	dbDEMC;HMDD V2.0
hsa-mir-134	dbDEMC	hsa-mir-132	dbDEMC;HMDD V2.0
hsa-mir-122	dbDEMC;HMDD V2.0	hsa-mir-218	dbDEMC;HMDD V2.0
hsa-mir-221	dbDEMC;miR2Disease; HMDD V2.0	hsa-mir-222	dbDEMC;miR2Disease; HMDD V2.0
hsa-mir-133a	dbDEMC;HMDD V2.0	hsa-mir-137	dbDEMC;HMDD V2.0
hsa-mir-196a	dbDEMC;miR2Disease; HMDD V2.0	hsa-mir-29b	dbDEMC;miR2Disease; HMDD V2.0
hsa-mir-7	dbDEMC;miR2Disease; HMDD V2.0 V2.0	hsa-mir-15b	dbDEMC
hsa-mir-34a	dbDEMC;HMDD V2.0	hsa-mir-20a	miR2Disease;HMDD V2.0
hsa-mir-125b	miR2Disease;HMDD V2.0	hsa-mir-96	dbDEMC;miR2Disease; HMDD V2.0
hsa-mir-16	dbDEMC;HMDD V2.0	hsa-mir-205	dbDEMC;miR2Disease; HMDD V2.0
hsa-mir-1	dbDEMC;HMDD V2.0	hsa-mir-200c	dbDEMC;miR2Disease; HMDD V2.0
hsa-mir-26a	dbDEMC;miR2Disease; HMDD V2.0	hsa-mir-326	dbDEMC;HMDD V2.0
hsa-mir-146a	dbDEMC;miR2Disease; HMDD V2.0	hsa-mir-34b	dbDEMC;HMDD V2.0
hsa-mir-29c	dbDEMC;miR2Disease; HMDD V2.0	hsa-mir-200a	dbDEMC;miR2Disease; HMDD V2.0
hsa-mir-223	dbDEMC;HMDD V2.0	hsa-mir-148a	dbDEMC;miR2Disease; HMDD V2.0
hsa-mir-206	dbDEMC;miR2Disease; HMDD V2.0	hsa-mir-29a	dbDEMC;HMDD V2.0
hsa-mir-142	unconfirmed	hsa-mir-302b	dbDEMC;HMDD V2.0
hsa-mir-9	dbDEMC;miR2Disease; HMDD V2.0	hsa-mir-34c	dbDEMC;HMDD V2.0
hsa-mir-21	dbDEMC;miR2Disease; HMDD V2.0	hsa-mir-30b	dbDEMC;HMDD V2.0
hsa-mir-200b	dbDEMC;miR2Disease; HMDD V2.0	hsa-mir-182	dbDEMC;miR2Disease; HMDD V2.0
hsa-mir-199a	dbDEMC;HMDD V2.0	hsa-mir-1207	unconfirmed
hsa-mir-224	dbDEMC;HMDD V2.0	hsa-mir-302a	dbDEMC;HMDD V2.0
hsa-mir-145	dbDEMC;miR2Disease; HMDD V2.0	hsa-mir-10b	dbDEMC;miR2Disease; HMDD V2.0
hsa-mir-124	dbDEMC;HMDD V2.0	hsa-mir-150	dbDEMC

Finally, to test robustness of our model, we carried out the third case study for Carcinoma Hepatocellular based on known associations in HMDD V1.0 including 1395 associations between 271 miRNAs and 137 diseases. In this case study, we ranked candidate miRNAs for Carcinoma Hepatocellular and validated top 50 predictions with experimental evidences. As has been defined, a candidate miRNA was a miRNA unassociated with the Carcinoma Hepatocellular according to HMDD v1.0, which guaranteed that validation of the predictions was completely independent of training database HMDD V1.0. As a result, 10 out of top 10 and 44 out of top 50 potential miRNAs associated with Carcinoma Hepatocellular were validated by HMDD V2.0, dbDEMC and miR2Disease (See Table 7). For example, hsa-mir-146b (1st in the prediction list) was down-regulated in Carcinoma Hepatocellular and could inhibit tumor growth and metastasis of Carcinoma Hepatocellular [62]. Aforementioned results indicate that EDTMDA has good robustness, showing satisfactory performance in different dataset.

**Table 7:** EDTMDA was implemented to predict potential miRNAs related to Carcinoma Hepatocellular based on known associations in HMDD V1.0 database. The top 50 predicted miRNAs were verified in dbDEMC, miR2Disease and HMDD V2.0. The first column records top 1–25 related miRNAs and the third column records the top 26–50 related miRNAs.

miRNA	evidence	miRNA	evidence
hsa-mir-146b	HMDD V2.0	hsa-mir-29a	dbDEMC;HMDD V2.0
hsa-mir-155	dbDEMC;miR2Disease; HMDD V2.0	hsa-mir-194	dbDEMC;miR2Disease
hsa-mir-128b	miR2Disease	hsa-let-7i	dbDEMC;HMDD V2.0
hsa-mir-106b	dbDEMC;miR2Disease; HMDD V2.0	hsa-mir-93	dbDEMC;miR2Disease; HMDD V2.0
hsa-mir-126	dbDEMC;miR2Disease; HMDD V2.0	hsa-mir-34b	unconfirmed
hsa-mir-143	dbDEMC;miR2Disease	hsa-mir-30c	miR2Disease;HMDD V2.0
hsa-mir-210	dbDEMC;HMDD V2.0	hsa-mir-429	unconfirmed
hsa-mir-141	miR2Disease;HMDD V2.0	hsa-mir-135b	unconfirmed
hsa-let-7a	dbDEMC;miR2Disease; HMDD V2.0	hsa-mir-15a	dbDEMC;miR2Disease; HMDD V2.0
hsa-mir-132	miR2Disease	hsa-mir-30d	dbDEMC;HMDD V2.0
hsa-mir-25	dbDEMC;miR2Disease; HMDD V2.0	hsa-mir-205	miR2Disease;HMDD V2.0
hsa-let-7g	miR2Disease;HMDD V2.0	hsa-mir-153	unconfirmed
hsa-mir-29b	dbDEMC;HMDD V2.0	hsa-mir-383	unconfirmed
hsa-mir-214	dbDEMC;miR2Disease; HMDD V2.0	hsa-mir-196b	unconfirmed
hsa-let-7d	miR2Disease;HMDD V2.0	hsa-mir-200c	HMDD V2.0
hsa-mir-181b	dbDEMC;miR2Disease; HMDD V2.0	hsa-mir-451	dbDEMC
hsa-mir-24	miR2Disease;HMDD V2.0	hsa-mir-219	miR2Disease;HMDD V2.0
hsa-let-7b	miR2Disease;HMDD V2.0	hsa-mir-7	HMDD V2.0
hsa-let-7f	miR2Disease;HMDD V2.0	hsa-mir-151	miR2Disease
hsa-let-7c	dbDEMC;miR2Disease; HMDD V2.0	hsa-mir-30e	miR2Disease
hsa-mir-9	miR2Disease	hsa-mir-192	miR2Disease;HMDD V2.0
hsa-mir-191	dbDEMC;HMDD V2.0	hsa-mir-103	miR2Disease
hsa-mir-16	dbDEMC;miR2Disease; HMDD V2.0	hsa-mir-26b	dbDEMC;miR2Disease
hsa-mir-29c	dbDEMC;HMDD V2.0	hsa-mir-218	HMDD V2.0
hsa-mir-34c	HMDD V2.0	hsa-mir-339	unconfirmed

### Label randomization test

We randomly shuffled ‘1’ and ‘0’ elements and kept their respective numbers unchanged in adjacency matrix, which was used to test whether our model suffered from overfitting. The AUC of three cross validations including global LOOCV, local LOOCV and 5-fold CV were 0.4939, 0.4413 and 0.5005+/-0.0029 respectively, which indicated that EDTMDA effectively avoided overfitting. Furthermore, label randomization test was implemented in three case studies by randomly shuffling ‘1’ and ‘0’ elements and keeping their respective numbers unchanged in adjacency matrix. The results were shown in Table 8, compared with the results under true labels. From the comparison results, we could draw the conclusion that EDTMDA is an effective tool to unveil more potential miRNAs related to diseases.

**Table 8:** The number of validated miRNAs among top 10 and top 50 predicted miRNAs in case studies between under true labels and under label randomization.

Case study	Top 10 & true labels	Top 10 & label randomization	Top 50 & true labels	Top 50 & label randomization
The 1st type of case study for Esophageal Neoplasms	10	4	47	26
The 1st type of case study for Kidney Neoplasms	9	5	43	22
The 2nd type of case study for Breast Neoplasms	10	5	48	36
The 3rd type of case study for Carcinoma Hepatocellular	10	5	44	33

### Different ways to select negative samples

In our model, we randomly selected some miRNA-disease pairs without known associations as negative samples. Moreover, considering that different diseases with different numbers of associated miRNAs, we designed a new way to select negative samples, which reflected the contribution of each disease to the positive sample set. For the new way, negative samples were sampled randomly for each disease to have the same size as the positive samples of the disease. That is, more negative samples were sampled for the disease with more known associated miRNAs. This new way to select negative samples was named local random and the previous way to select negative samples from all the negative was named global random. For the model using local random to select negative samples, we implemented model evaluation under three cross validations (global LOOCV, local LOOCV and 5-fold CV), and the AUCs were 0.8224, 0.7871 and 0.8180+/-0.0019 respectively, which was significantly inferior to AUCs of 0.9309, 0.8524 and 0.9192+/-0.0009 in our model using global random to select negative samples. For the local random to select negative samples, the poor performance of model could be that more false negative samples (miRNA-disease pairs with potential associations) were selected. It is apparently observed that miRNAs prefer to relate to some specific diseases in our dataset and we think that there should be more potential miRNA-disease associations for these specific diseases. But in local random to select negative samples, more selected negative samples were derived from the negative of those specific diseases with more related miRNAs, i.e., more false negative samples were selected. In global random to select negative samples, we avoided selecting more false negative samples for model training and obtained better model performance.

## Discussion

Increasing researchers are devoted to developing computational methods to infer potential miRNA-disease associations as these methods can be valuable complements to experiments. In this study, we proposed a computational method called EDTMDA under the framework of ensemble learning and dimensionality reduction. The Gaussian interaction profile kernel similarity scores for miRNAs and diseases were first calculated from known miRNA-disease associations. Then integrated miRNA (disease) similarity could be obtained via integrating miRNA functional similarity (disease semantic similarity) and Gaussian interaction profile kernel similarity of miRNAs (diseases). In addition, the feature vectors for the miRNA-disease pair was constructed by conducting feature extraction on integrated similarity and known miRNA-disease associations. Multiple base learnings were built based on random selection of negative samples and miRNA/disease features so that many decision trees (DTs, base classifiers) were attained. Particularly, in order to remove the noise or redundancy, PCA was utilized to reduce feature dimensionality during each base learning. Final prediction results were given by adopting simple average strategy for these DTs.

The success of this model is mainly due to the following points. First, comprehensive statistical features, graph theoretic features and matrix factorization results were extracted from similarity information and known associations so that informative input features for the model could be obtained. Furthermore, because feature profiles made the most of similarity and known associations, EDTMDA could work for new diseases without known association information. Second, ensemble learning was designed to integrate multiple basic classifiers for more accurate prediction. In addition, feature dimensionality reduction with PCA could remove noise or redundancy to further improve prediction performance. Third, for the base classifier, the regression tree model with the arithmetic of Classification and Regression Tree (CART) was selected in our model, which was the binary tree with simple structure and could avoid the data fragmentation existing in multi-branching tree.

However, there were several limitations in our prediction model. To begin with, known miRNA-disease associations were inadequate (with only 2.86% of 189,585 miRNA-disease pairs being labeled) and increasing associations confirmed by experiments in the future would further improve model performance. Additionally, similarity calculation of miRNA and disease in this study may not be perfect and we expect more biological information would be incorporated into similarity measurement. Moreover, EDTMDA might cause bias to miRNAs which have more associated disease records. Finally, negative samples (miRNA-disease pairs without associations) were needed in our model. We randomly sampled some pairs without known associations as negative samples for model training. In order to reduce bias and improve prediction performance, multiple base classifiers were trained and integrated. Moreover, dimensionality reduction was employed for each base classifier to reduce noise and redundant information, which further improve performance of model. Actually, it is still difficult to obtain true negative samples (That is, miRNA-disease pairs show no evidence of association), because these true negative samples are scarcely reported in literature. We will make efforts to develop the new approach to identify reliable negative samples in the future.

## Supporting information

S1 Table We applied EDTMDA to prioritize all the candidate miRNA-disease pairs based on all the known miRNA-disease associations in HMDD V2.0 database as training samples. This prediction result is released for further experimental validation and research.
(XLSX)
